# Dietary and Nutritional Guidelines for People with Diabetes

**DOI:** 10.3390/nu15204314

**Published:** 2023-10-10

**Authors:** Katsumi Iizuka, Daisuke Yabe

**Affiliations:** 1 Department of Clinical Nutrition, Fujita Health University, Toyoake 470-1192, Japan; 2 Food and Nutrition Service Department, Fujita Health University Hospital, Toyoake 470-1192, Japan; 3 Department of Diabetes, Endocrinology and Metabolism, Gifu University Graduate School of Medicine, Gifu 501-1194, Japan; yabe.daisuke.s9@f.gifu-u.ac.jp; 4 Department of Rheumatology and Clinical Nutrition, Gifu University Graduate School of Medicine, Gifu 501-1194, Japan; 5 Center for One Medicine Innovative Translational Research, Gifu University, Gifu 501-1194, Japan; 6 Yutaka Seino Distinguished Center for Diabetes Research, Kansai Electric Power Medical Research Institute, Kyoto 604-8436, Japan

Diabetes is a disease in which lifestyle-based interventions, including recommendations for a healthy diet, play a critical role, and many countries have established their own nutritional guidelines. Common to all countries is a commitment to individualized nutrition therapy according to personal preferences and treatment goals. However, it is impossible to standardize nutritional therapy because body size, age, lifestyle including diet, and national characteristics vary from country to country and even from region to region within the same country. However, by reading guidelines from different countries, one can learn about different national attitudes toward food. Many may find it useful to learn about these different ways of thinking about diet therapy in other countries.

Canada is a multiethnic country with people who have many different cultural backgrounds. Many patients with diabetes in Canada are obese, which differs from patients in Asia [1]. Nutritional guidance from Diabetes Canada recommends eating according to Canada’s healthy eating guide for the general population [1]. This guide is designed to help people consume their daily protein, carbohydrates, vitamins, and minerals from a variety of foods in four categories (vegetables and fruits, grains, milk and alternatives, and meat and alternatives). Because 80–90% of patients with diabetes in Canada are diagnosed as obese, the emphasis is on weight loss through total energy control; thus, the total energy level should be appropriate for weight management (no numerical target). In adults with diabetes, the macronutrient distribution as a percentage of total energy can range from 45% to 60% carbohydrate, 15% to 20% protein, and 20% to 35% fat to allow for individualized nutrition therapy according to preferences and treatment goals. To reduce the risk of cardiovascular diseases, adults with diabetes should avoid trans fatty acids and should consume less than 9% of total daily energy from saturated fatty acids, replacing these fatty acids with polyunsaturated fatty acids (PUFAs), particularly mixed *n* − 3/*n* − 6 sources, monounsaturated fatty acids (MUFAs) from plant sources, whole grains, or carbohydrates with a low glycemic index (GI). Adults with diabetes may substitute added sugars for other carbohydrates as part of mixed meals up to a maximum of 10% of total daily energy intake, provided adequate control of blood glucose, lipids, and body weight is maintained. Adults with type 1 and type 2 diabetes should aim to consume 30 to 50 g/day of dietary fiber, with a third or more (10 to 20 g/day) coming from viscous soluble dietary fiber to improve glycemic control and low-density lipoprotein cholesterol and reduce the cardiovascular risk. Dietary intervention methods include macronutrient-based approaches (low-GI diet, high-fiber diet, high-MUFA diet, low-carbohydrate diet, high-protein diet), Mediterranean dietary patterns, alternative diets (e.g., vegetarian, Dietary Approaches to Stopping Hypertension (DASH) diet, Portfolio diet, Nordic diet, Atkins diet), dietary patterns involving specific foods (dietary pulses/legumes, fruits and vegetables, nuts, whole grains, dairy), and meal replacement, as introduced in the guidelines.

The American Diabetes Association (ADA) Consensus Report clearly states that one-size-fits-all meal plans have no evidence for diabetes prevention and stresses the importance of individualization [2]. Furthermore, the report clearly states that medical nutrition therapy (MNT) is the foundation of all diabetes management. Furthermore, MNT should be provided in response to diabetes medication and activity changes. The ADA suggests that an ideal carbohydrate/fat/protein ratio does not exist and should, therefore, be set individually. A minimum fiber intake of 14 g per 1000 calories is recommended. Regarding eating patterns, it is important to combine a variety of foods. In particular, it is recommended to avoid added sugars and refined grains and to choose whole foods instead of ultra-processed foods. Additionally, because most evidence shows that reducing the total amount of carbohydrates improves blood glucose, it is acceptable to choose a dietary pattern (e.g., Mediterranean diet) that is appropriate for each individual. It is also recommended that sugar-sweetened beverages should be replaced with water. The adverse effects of artificial sweeteners, such as reduced awareness of energy intake, are also described. There is no supporting evidence for vitamin or trace element supplementation, so it is generally not recommended, but the measurement of vitamin B12 is recommended for patients taking metformin. Finally, in terms of preventing complications, it is recommended to reduce carbohydrates, switch from foods containing saturated fatty acids to foods containing unsaturated fatty acids, limit salt, and consume fish twice a week. It has been stated that protein restriction (0.8 g/kg BW/day) for diabetic kidney disease (DKD) may actually increase the risk of undernutrition and sarcopenia. In diabetic gastroparesis, reducing portion sizes may improve symptoms. The correction of hyperglycemia is also mentioned as an option. Individualized nutritional approaches are described. However, approaches that examine genetic predisposition, metabolites, and gut bacteria have yet to be considered factors that improve outcomes.

The European Association for the Study of Diabetes published a recommendation for diet therapy in 2023. A combination of dietary patterns (e.g., Mediterranean, Nordic, vegetarian) and physical activity is proposed, similar to the Canadian guidelines [3]. Energy and physical activity levels are recommended in amounts that maintain a healthy body weight over the long term. The emphasis on weight loss for obesity is different from that in Japan, where there are many older people. Dietary replacements are also mentioned. The recommendation clearly states that too many carbohydrates and low-carbohydrate ketogenic diets are not recommended for weight loss. It is stated that dietary fiber should be taken at a minimum of 35 g/day, and fiber supplements should be considered when diet alone is insufficient. A low-GI diet is also recommended. Carbocounting for determining insulin levels in type 1 diabetes may also be useful. Dietary lipids should come from plant-based foods that contain MUFAs and PUFAs. Saturated and trans fatty acids should be limited to 10% and 1% of total energy, respectively. Similar to Japan, protein intake is also determined on the basis of kidney function and age in Europe. For individuals with normal renal function and those under or over 65 years of age, 10–20% and 15–20% of total energy intake is recommended, respectively. In the case of obesity and normal renal function, a short-term protein intake of 23–32% is acceptable; a total of 10–15% protein intake is recommended for stage 3a nephropathy. As in Canada, a plant-based diet is also suggested, in which whole grains, whole fruits and vegetables, legumes, nuts, and seeds are recommended. Furthermore, the Mediterranean diet, Nordic dietary pattern, and vegetarian dietary pattern are recommended. Most importantly, the European guidelines address ultra-processed foods. Of particular importance is the recommendation that processed meats, red meats, sugar-sweetened beverages, and refined grains should be reduced, and foods of plant origin, which are less processed, should be increased. However, there are no quantitative indicators, and there is no mention of vitamins or trace elements.

The number of older people in Japan is increasing more than in any other developed country, and the concept of nutritional therapy, especially for older individuals, is discussed in detail [4]. First, target weights are set for each age group (under 65 years, 65–74 years, and 75 years or older), and total energy is calculated from energy coefficients for each physical activity level. However, this is only one guideline, and it is determined according to the condition here. In particular, frailty, complications, body composition, height reduction, eating status, and nutritional status must be taken into consideration in individuals aged 75 years and older. There is no clear evidence establishing a desirable ratio of energy-producing nutrients for the prevention and management of diabetes, so it is necessary to individualize the intake. However, as a general guideline, it is stated that carbohydrates should account for 50–60% of energy (150 g/day or more), protein should be 20% of energy or less, and the rest should be lipids. However, if lipids exceed 25% energy, PUFAs should be increased. Dietary fiber is suggested to be at least 20 g/day, and sucrose should be avoided (no amount is given). The Japanese consensus statement is characterized by attention to diabetic nephropathy and older people with respect to protein intake. Although low-protein diets are often used to treat kidney disease, it is recommended that protein intake should be individually set in low-protein diets among older patients with sarcopenia, frailty, or who are at risk for such conditions. The total energy intake should be no less than 0.8 g/kg BW/day and 30–35 kcal/kg BW/day. Even if a low-protein diet is not implemented, it is recommended that the total energy intake be no less than 1.3 (1.5) g/kg BW/day. With regard to dietary interventions, guidance on regular meal intake and the order of eating (staple foods are eaten last) is presented.

It is interesting to note that the emphasis differs between Japan, where the focus is the older population, and consensus reports in Canada, the United States, and Europe where there is a focus on patients with obesity-based diabetes. In terms of dietary intervention, the ADA, Europe, and Canada recommend dietary patterns, such as the Mediterranean diet, Nordic dietary pattern, and vegetarian dietary pattern, and the emphasis is on heart disease prevention. In Japan, however, emphasis is placed on protein intake to prevent sarcopenia and frailty in older individuals. Nutritional therapy for DKD is also recommended in the United States, Japan, and Europe.

A common problem arising in many areas is that of ultra-processed foods and artificial sweeteners [5,6,7,8,9,10,11,12,13,14]. The consumption of ultra-processed foods is associated with obesity and cancer risk [5,6,7,8]. Ultra-processed foods are high in saturated fats and added sugars and low in vitamins and fiber. In particular, many guidelines make little mention of vitamin and mineral sufficiency goals. Vitamin deficiencies may appear in older patients with dental problems or problems with gastrointestinal function. It should be noted that drugs, such as antacids and metformin, may interfere with vitamin B12 absorption [15]. The ADA guidelines recommend annual B12 measurements in patients taking metformin. Vitamin D deficiency is also common, and some guidelines recommend supplementation in older individuals. Additionally, the accumulation of carcinogens (Maillard reaction products such as acrylamide and nitroso compounds derived from nitrous acid) is a major problem. The question of what proportion of ultra-processed foods should be included in the daily diet is also expected to become a focal point. The issue of artificial sweeteners is also a concern [9,10,11,12,13,14]. Recent guidelines from the World Health Organization state that long-term use of artificial sweeteners increases the risk of diabetes, obesity, and cancer [9,10,11,12,13,14]. Some believe that replacing sugar with artificial sweeteners is effective for weight loss, but an increased cancer risk has also been reported, especially related to obesity [9,10,11,12,13,14]. Many guidelines mention artificial sweeteners but are unclear; thus, further evidence is needed in patients with diabetes.
